# Safety and efficacy of biologic immunosuppressive treatment in juvenile idiopathic arthritis associated with inborn errors of immunity

**DOI:** 10.3389/fped.2024.1353825

**Published:** 2024-02-26

**Authors:** V. Accardo, I. Pagnini, I. Maccora, E. Marrani, M. V. Mastrolia, G. Simonini

**Affiliations:** Rheumatology Unit, Meyer Children's Hospital IRCCS, Florence, Italy

**Keywords:** arthritis, juvenile, inborn errors of immunity, DiGeorge syndrome, Bruton-type agammaglobulinemia, adalimumab, etanercept, tocilizumab

## Abstract

**Objectives:**

This study aims to describe clinical features, therapeutic outcomes, and safety profiles in patients affected by juvenile idiopathic arthritis (JIA) and inborn errors of immunity (IEI) treated with biological Disease-modifying antirheumatic drugs (DMARDs).

**Methods:**

We enrolled three patients who were followed in the Pediatric Rheumatology Unit at Meyer Children's Hospital in Florence; these patients were affected by JIA, according to ILAR criteria, and IEI, according to the IUIS Phenotypical Classification for Human Inborn Errors of Immunity. Among them, two patients had 22q11.2 deletion syndrome (22q11.2DS) and one patient had X-linked agammaglobulinemia (XLA).

**Results:**

Case 1: A 6-year and 2-month-old boy was affected by 22q11.2DS, associated with oligoarticular JIA, at the age of 2 years. He was treated with non-steroidal anti-inflammatory drugs (NSAIDs) and methotrexate, along with oral glucocorticoids but with no benefits. Treatment with etanercept allowed him to achieve remission after 10 months. Case 2: A 6-year and 2-month-old girl was affected by 22q11.2DS, associated with oligoarticular JIA, at the age of 3 years and 11 months. She was treated with NSAIDs, joint injections, and methotrexate but without clinical response. Treatment with Adalimumab allowed her to achieve remission after 6 months. Case 3: A 12-year and 2-month-old boy was affected by XLA, associated with polyarticular JIA, at the age of 9 years and 11 months. He was treated with NSAIDs, methotrexate, joint injections, and oral glucocorticoids with no benefits. He failed to respond to anti-TNF-alpha, tocilizumab, and abatacept. Currently, he is undergoing therapy with sirolimus plus abatacept, which allowed him to achieve remission after 4 months.

**Conclusions:**

Results suggest that the use of immunosuppressive biological therapies can control disease activity in these patients. No adverse drug-related reactions were observed during the follow-up.

## Introduction

Inborn errors of immunity (IEI) are a heterogeneous group of diseases affecting different components of the immune system. Overall, patients with IEI have an increased susceptibility to infectious diseases and autoimmune diseases, including juvenile idiopathic arthritis (JIA) (1). In 22q11.2 deletion syndrome (22q11.2DS), previously known as DiGeorge syndrome, a congenital chromosome deletion syndrome (22q11.2 del) characterized by decreased T-cell numbers secondary to thymic hypoplasia, JIA occurs 50 times more commonly than in the normal population (2). In X-linked agammaglobulinemia (XLA), a genetic disorder resulting in maturational disturbance of B-cell development due to a mutation in Bruton's tyrosine kinase (BTK), JIA has a prevalence of about 13% (3). We report a case series of three patients with IEI, receiving different immunosuppressive treatments due to the severe active disease of concomitant JIA.

## Materials and methods

To be eligible for the case series description, patients should fulfill the diagnosis criteria of JIA, according to the ILAR criteria (4), and the diagnosis criteria of IEI, according to the IUIS (International Union of Immunological Societies) Phenotypical Classification for Human Inborn Errors of Immunity (5). Clinical charts of eligible patients were retrospectively reviewed at the disease onset and during the disease course. The disease onset of the first eligible patient was documented in August 2019. Over the period between January 2019 and December 2023, three patients fulfilled the inclusion criteria of the study. During the same time frame, 750 children with JIA were currently being followed at our unit.

## Results

Case 1: A boy affected by 22q11.2DS, at the age of 2 years and 3 months, developed arthritis in the right ankle. MRI of the right ankle revealed synovitis and concomitant effusion in joint spaces. A diagnosis of oligoarticular JIA was made, and treatment with non-steroidal anti-inflammatory drug (NSAIDs) and methotrexate (MTX, dose 15 mg/mq s.c. once a week) was started. After 6 months with MTX, he complained of persistent arthritis in the right ankle, therefore leading to a steroid joint injection. One month later, the patient experienced another flare-up in the right ankle, and treatment with etanercept (0.8 mg/kg/week) was then started. Nonetheless his mono-articular course, the patient achieved a stable and persistent remission only after 10 months from starting Etanercept, while also maintaining MTX treatment. After at least 2 years of persistent remission, we attempted to wean and stop MTX over an additional 1 year. At the last follow-up, at the age of 6 years and 2 months, he is still in remission on etanercept and has started to wain this treatment. Over the course of JIA treatment with immunomodulatory drugs, the boy did not experience an increased rate of infections or serious adverse events.

Case 2: A girl affected by 22q11.2DS, at the age of 3 years and 6 months, developed left knee arthritis. MRI of the involved joint revealed signs of synovial thickening and joint effusion. She received NSAIDs for 16 weeks with no clinical response. Eight months later, a diagnosis of oligoarticular JIA was made, which was associated with severe uveitis, cataract, and iridolenticular synechia. She was treated with joint injections and MTX (dose 15 mg/mq s.c. once a week), but did not show improvement. After 8 months of treatment, MTX was stopped due to an increase in liver enzyme, and adalimumab (20 mg/2 weeks) was then started. After 6 months, the patient achieved clinical remission. At 5 years and 4 months, while undergoing therapy with adalimumab, she developed arthritis in the left knee, and by the age of 5 years and 11 months, she developed arthritis in both knees, leading to treatment with joint injections. At the last follow-up, at 6 years and 1 month, she is still being treated with adalimumab (increased to 40 mg/2 weeks for weight >30 kg), achieving clinical remission as regards arthritis and uveitis. The ocular condition remains stable, except for the ocular cataract, which was surgically treated. Over the course of Adalimumab treatment, no cases of infection or other adverse drug-related reactions were observed. Anti-adalimumab antibodies were periodically checked and yielded negative results (ELISA testing).

Case 3: A boy with XLA, in treatment with subcutaneous immunoglobulins since he was 2 years old, developed arthritis in the left knee at the age of 9 years and 11 months. After a few months, he developed arthritis in both ankles, knees, and elbows, as well as in I and V proximal interphalangeal (PIP) joints of the left hand, along with enthesitis of the right peroneal, tibial, and Achilles tendons. MRI scanning of both knees was performed, revealing signs of synovitis and modest joint effusion. Upon the diagnosis of polyarticular JIA, treatment with NSAIDs, MTX, and oral steroids was started. After 3 months, he developed new arthritis in both wrists and I and III PIP joints of the right hand. So, adalimumab (20 mg/2 weeks) was started, in addition to oral steroids for a month. Two months later, he developed arthritis in the knees and left ankle; thus, he received multiple joint injections. At the age of 10 years and 8 months, adalimumab was discontinued due to disease progression, and etanercept (0.8 mg/kg/weekly) was started, but without efficacy. Four months later, etanercept was switched to tocilizumab (8 mg/kg every 4 weeks), but arthritis persisted in both ankles, knees, and wrists. At the age of 11 years and 6 months, tocilizumab was switched to abatacept (10 mg/kg every 4 weeks) due to poor response. Despite additional joint injections for persistent arthritis in both knees, wrists, elbows, and PIP joints, his polyarthritis disease persisted. When he was 12 years old, sirolimus was added to the treatment regimen with abatacept and oral steroids, therefore discontinuing MTX, considering the possible role of mTOR protein in modulating immune response and influencing the production of inflammatory cytokines (6). Four months later, he achieved clinical remission, and steroids were then weaned. No cases of infection or serious adverse reactions were observed during treatment. In our cohort, no other autoimmune diseases, infections, or lymphoproliferative disorders were reported. In addition, to evaluate disease remission, the Juvenile Arthritis Disease Activity Score (JADAS) and Childhood Health Assessment Questionnaire (CHAQ) were scored. A summary of the demographic, clinical, laboratory, and therapeutic data of all three patients is presented in Table 1.

**Table 1:** Demographic data, laboratory tests, clinical features, diagnostic data, therapeutic data, and outcomes at the last follow-up.

No.	Gender	Age at IEI diagnosis	Clinical features	Diagnosis of IEI	Age at JIA diagnosis	Lab tests at JIA diagnosis	Immunological tests at JIA diagnosis and during disease course	Joint involvement at JIA diagnosis	Eye involvement	Age at the start of MTX	Time on MTX	Age at the start of bDMARDs	Type and duration of bDMARDs	Other treatments	Number and location of joint injections	Duration of the follow-up	Age at the last follow-up	Clinical outcomes at the last follow-up
1	M	1 month	Heart malformations, recurrent infections, hypocalcemia	22q11.2 del and dup (17) (p11.2p11.2)	2 years 3 months	↑ CRP and ESR, ANA and pANCA positivity, ASCA and cANCA negativity	Normal serum Ig, normal lymphocyte subtypes, normal DHR, pathological MBC, normal DNT, normal TREC, normal KREC, normal RTE	R ankle	—	2 years 5 months	45	2 years 11 months	ETA (38 m)	—	1: L ankle	46	6 years 1 month	Clinical remission on ETA
2	F	2 months	Labio-palatal schisis, auricular bilateral appendix, heart malformations, delayed growth, intellectual disability	22q11.2 del	4 years 2 months	↑ CRP, normal ESR, ANA negativity	↑ serum Ig, ↓ CD4, normal DHR, pathological MBC, normal DNT, normal TREC, normal KREC, normal RTE	L knee	Uveitis, cataract, iridolenticular synechia	4 years 2 months	8	4 years 10 months	ADA (16 m)	—	2: L knee twice	25	6 years 2 months	Clinical remission on ADA
3	M	2 years	Recurrent infections	IVS14+1 G>A	9 years 11 months	↑ CRP, normal ESR, ANA negativity, HLA B27 negativity	Normal serum IgG, IgA and IgM deficiency, DHR, pathological MBC, normal TREC, normal KREC, normal DNT, normal RTE	R and L ankles, elbows and knees, I and V R PIP, enthesitis of the R peroneal, tibial, and Achilles tendons	—	10 years	24	10 years 2 months, 10 years 8 months, 11 years, 11 years 6 months	ADA (6 m), ETA (4 m), TOC (6 m), ABA (7 m)	ScIg^a^ Sir^b^	Multiple joint injections^c^	29	12 years 4 months	Clinical remission on ABA and Sir

ABA, abatacept; ADA, adalimumab; CCS, corticosteroid; CRP, C-reactive protein; DHR, dihydrorhodamine; DNTs, CD3^+^CD4^−^CD8^−^ double negative T-cells; ESR, erythrocyte sedimentation rate; ETA, Etanercept; F, female; IEI, inborn errors of immunity; JIA, juvenile idiopathic arthritis; KREC, K deleting receptor excision circle; L, left; M, male; MBCs, memory B cells; R. right; RTE, CD4 T-cell recent thymic emigrant; ScIg, subcutaneous immunoglobulin; Sir, Sirolimus; TOC, Tocilizumab; TREC, T-cell receptor excision circle.

Demographic data include gender, age at IEI diagnosis, age at JIA diagnosis, age at the start of MTX treatment, age at the start of bDMARDs treatment, age at the last follow-up expressed in years and months, and total duration of follow-up expressed in months; laboratory tests include inflammation markers such as CRP and ESR, autoimmunity data such as ANA, cANCA, pANCA, ASCA, HLAB27 when performed, immunological data such as lymphocyte subtypes and serum Ig levels when performed, DHR test, DNT levels, MBC values, RTE values, TREC, and KRE; clinical features include IEI, joints involvement at JIA diagnosis and ocular involvement if present; diagnostic data include type of genetic test used to confirm the diagnosis of IEI: FISH analysis of 22q11.2 deletion for DiGeorge syndrome, sequence analysis of exons 1–19 of BTK gene for X-linked agammaglobulinemia; and therapeutic data include duration of MTX treatment expressed in months, type and duration of bDMARD treatment expressed in months, other treatments and their duration expressed in months, number and location of joint injections.

^a^ ScIg were introduced at the age of 2 yrs for agammaglobulinemia due to the underlying disease (XLA).

^b^ Sirolimus was added at the age of 12 yrs old. Current duration of treatment is 2 mo.

^c^ Joint injections were performed in two occasions: the first knees and L ankle; the second time elbows and knees.

## Discussion

The association between autoimmune diseases and inborn errors of immunity is well described in the literature, but poor evidence is available regarding the treatment outcomes. JIA is an antigen-driven autoimmune process, involving several mediators such as lymphocyte Th1, B cells, and macrophages (7). Autoimmune diseases have been associated with 22q11.2DS, probably linked to T-cell regulatory defects and impaired central tolerance. In XLA, low IgG levels, in addition to increasing susceptibility to infections, may be the basis of the inflammatory mechanism of JIA. Furthermore, BTK gene is associated with the Toll-like receptor (TLR) pathway, whose activation leads to the production of cytokines, notably TNF (1, 8).

Disorder of immune regulation can be the basis of autoimmunity and could be an initial or predominant manifestation of certain IEIs. Growing attention is being directed toward exploring potential predictors of immune dysregulation and autoimmunity in IEI. For example, in 22q11.2DS, low CD3+ T-cell counts, B-cell dysfunction, and consequently low serum levels of immunoglobulin can increase the rate of developing autoimmunity, manifesting as immune cytopenias as well as hypothyroidism, hyperthyroidism, diabetes mellitus, psoriasis, vitiligo, alopecia, and rheumatologic disease including juvenile idiopathic arthritis and systemic sclerosis (9). Although no studies specifically focus on JIA, this could represent an interesting perspective.

In our experience, JIA in IEI seems to exhibit a more severe disease course, with recurrent arthritis, the need for several joint injections and different corticosteroid courses, and, according to the literature, poor response to traditional immunosuppressive drugs (2, 10–14). Conversely to the literature, in patient #3, the immunoglobulin substitutive therapy did not change the course of arthritis (13). The use of biologic Disease-modifying antirheumatic drugs (bDMARDs) in patients with IEI remains controversial. Brief comparison of different treatments in patients with JIA and IEI in literature is presented in Table 2. Although mandatory due to the activity of the disease and its progression, their use is not free of side effects, including increased susceptibility to opportunistic infections.

**Table 2:** Treatment of juvenile idiopathic arthritis and inborn errors of immunity.

Reference	Number of patients	IEI	Type of JIA	Treatment	Outcome	Side effects
Davies et al. (10)	5	22q11.2DS	#1 polyarticular	#1 joint injection + MTX + CCS	#1 efficacious	#1 N/A
			#2 polyarticular	#2 NSAID + HCQ + MTX	#2 efficacious	#2 N/A
			#3 polyarticular	#3 CCS + MTX	#3 efficacious	#3 N/A
			#4 polyarticular	#4 NSAID + CCS + MTX	#4 partial response	#4 N/A
			#5 polyarticular	#5 NSAID + MTX	#5 partial response	#5 N/A
Rasmussen et al. (11)	2	22q11.2DS	#1 polyarticular	#1N/A	#1 N/A	#1 N/A
			#2 oligoarticular	#2 NSAID	#2 partial response	#2 N/A
Sullivan et al. (12)	3	22q11.2DS	#1 polyarticular	#1 N/A	#1 N/A	#1 N/A
			#2 oligoarticular	#2 NSAID	#2 efficacious	#2 N/A
			#3 polyarticular	#3 NSAID + CCS + MTX	#3 partial response	#3 N/A
Patiroglu et al. (13)	2	XLA	#1 polyarticular	#1 SSZ + MTX + ETA + IVIG	#1 efficacious (IVIG)	#1Guillain–Barre syndrome (ETA)
			#2 polyarticular	#2 MTX + IVIG	#2 efficacious (IVIG)	#2
Vancsa et al. (14)	2	XLA	#1 polyarticular	#1 IVIG + NSAID + SSZ	#1 efficacious	#1 N/A
			#2 polyarticular	#2 IVIG + NSAID + SSZ	#2 N/A	#2 N/A

22q11.2DS, 22q11.2 deletion syndrome; CCS, corticosteroid; ETA, Etanercept; HCQ, hydroxychloroquine; IVIG, intravenous immunoglobulin; N/A, not assessed; SSZ, sulfasalazine; XLA, X-linked agammaglobulinemia.

In our cohort, bDMARDs were added to the treatment regimen of all patients achieving clinical remission after several months and different attempts. Patient 1 received Etanercept plus MTX, while patient 2 received adalimumab and several joint injections, with good clinical control. Patient 3 had not responded to anti-TNF-alpha, tocilizumab, abatacept, and several joint injections, but showed good clinical response to sirolimus after 2 months. Apart from JIA associated with IEI, TNF-alpha inhibitors have been used with good clinical efficacy in patients with 22q11.2DS with associated IBD and in one patient with XLA with associated pyoderma gangrenosum (8–16).

In terms of safety, literature data suggest that biological therapies are generally well-tolerated, with common side effects that typically do not necessitate drug discontinuation. However, severe adverse events, including serious infections, have been observed, particularly when biological therapies are used in conjunction with other immunosuppressive drugs such as corticosteroids and methotrexate (17).

In our cohort, treatment with biological immunosuppressors over a follow-up of at least 16 months (range 16–38 months) did not show significant adverse events, including severe infections. None of the used bDMARDs were stopped due to adverse events, but rather due to no efficacy.

In our experience, biological DMARDs have been shown to be helpful and safe in controlling disease activity in these patients. Notwithstanding, the use of these kinds of therapies in this cohort of patients needs to be further studied, and regular follow-ups are necessary for monitoring the possible side effects of the treatment.

## Data Availability

The original contributions presented in the study are included in the article/Supplementary Material; further inquiries can be directed to the corresponding author.
